# Development of BOLD signal hemodynamic responses in the human brain

**DOI:** 10.1016/j.neuroimage.2012.06.054

**Published:** 2012-11-01

**Authors:** Tomoki Arichi, Gianlorenzo Fagiolo, Marta Varela, Alejandro Melendez-Calderon, Alessandro Allievi, Nazakat Merchant, Nora Tusor, Serena J. Counsell, Etienne Burdet, Christian F. Beckmann, A. David Edwards

**Affiliations:** a Centre for the Developing Brain, MRC Clinical Sciences Centre, Imperial College London, Hammersmith Hospital, Du Cane Road, London, W12 0NN, UK; b Department of Neonatal Medicine, Imperial College Healthcare NHS Trust, Hammersmith Hospital, Du Cane Road, London, W12 0NN, UK; c Imaging Physics, Imperial College London, Hammersmith Hospital, Du Cane Road, London, W12 0NN, UK; d Department of Bioengineering, Imperial College London, South Kensington Campus, London SW7 2AZ, UK; e Donders Centre for Cognitive Neuroimaging, Radboud University, Kapittelweg 29, 6525 EN Nijmegen, The Netherlands; f MIRA Institute for Biomedical Technology and Technical Medicine, University of Twente, 7500 AE Enschede, The Netherlands

**Keywords:** Brain development, Neonate, Functional MRI, Hemodynamic response function

## Abstract

In the rodent brain the hemodynamic response to a brief external stimulus changes significantly during development. Analogous changes in human infants would complicate the determination and use of the hemodynamic response function (HRF) for functional magnetic resonance imaging (fMRI) in developing populations. We aimed to characterize HRF in human infants before and after the normal time of birth using rapid sampling of the Blood Oxygen Level Dependent (BOLD) signal. A somatosensory stimulus and an event related experimental design were used to collect data from 10 healthy adults, 15 sedated infants at term corrected post menstrual age (PMA) (median 41 + 1 weeks), and 10 preterm infants (median PMA 34 + 4 weeks). A positive amplitude HRF waveform was identified across all subject groups, with a systematic maturational trend in terms of decreasing time-to-peak and increasing positive peak amplitude associated with increasing age. Application of the age-appropriate HRF models to fMRI data significantly improved the precision of the fMRI analysis. These findings support the notion of a structured development in the brain's response to stimuli across the last trimester of gestation and beyond.

## Highlights

► First systematic characterization of the BOLD signal HRF in human neonates. ► A maturational trend in HRF morphology was identified. ► Application of empirical HRF models significantly improved neonatal fMRI analysis.

## Introduction

In the third trimester of gestation and the first months of postnatal life, the human brain undergoes a dramatic but structured sequence of maturation resulting in the establishment of the cortex and its associated framework of structural and functional connectivity (de Graaf-Peters and Hadders-Algra, 2006; Rutherford, 2001). The importance of this period is highlighted by the marked increase in neurological dysfunction seen in children born preterm (less than 37 weeks gestation), where untimely exposure to ex-uterine factors apparently interferes with neural development such that brain structure and function are adversely affected throughout later life (Volpe, 2009). Although multi-modal neuro-imaging studies in immature animals have begun to characterize the biochemical and neurophysiological processes underlying the establishment of functional neural activity, largely due to their invasive nature, these studies have not been performed in-vivo on human subjects (Blankenship and Feller, 2010; Corlew et al., 2004; Felleman and Van Essen, 1991; Harris et al., 2011).

Blood Oxygen Level Dependent (BOLD) contrast functional Magnetic Resonance Imaging (fMRI) is non-invasive and can identify patterns of activation across the whole brain (Kwong et al., 1992; Ogawa et al., 1992). Developmental studies of the early rat brain have found that positive BOLD contrast responses can be elicited from around postnatal day 11–13, which equates to approximately 28–32 weeks in human gestation (Colonnese et al., 2008). Increasing age is characterized by a systematic increase in the peak amplitude of BOLD responses, larger and more widespread responses, and co-activation of the ipsilateral cortex and supplementary areas in addition to the primary sensory areas (Chan et al., 2010; Colonnese et al., 2008). In addition, coupled local field potential (LFP) recordings and BOLD contrast have also been found to show a progressive decrease in the time to peak response with increasing age (Colonnese et al., 2008). Developmental increases in the upregulation of carbonic anhydrase activity were found to be fundamental to the observed maturational trends, suggesting that resting cerebral blood flow (CBF) control plays a key role in these changes (Colonnese et al., 2008).

fMRI has not been widely applied to the human neonatal population due to a history of methodological challenges and inconsistent results (Seghier et al., 2006). Central to these difficulties has been the uncertainty about the polarity of the elicited functional responses, with some studies suggesting that in contrast to the canonical adult response, sensory stimulation induces a decrease in BOLD signal (often termed ‘negative BOLD’) during a transitory developmental stage in early infancy (Born et al., 2000; Heep et al., 2009; Seghier et al., 2004; Yamada et al., 2000). This ambiguity is further compounded by the use of sedative medication and/or anesthesia, which is often necessary for neonatal subjects during MRI examination to reduce distress and motion artifact (Marcar et al., 2006; Seghier et al., 2006).

The apparent inconsistencies in fMRI studies during the newborn period could be caused by developmental differences in the hemodynamic response to stimulation. In the adult human brain, the morphology of the Hemodynamic Response Function (HRF) has been well characterized and found to be reproducible and consistent across different populations (Aguirre et al., 1998; Glover, 1999; Handwerker et al., 2004), but even subtle inter-subject HRF variability has been found to significantly affect the identification of functional activity (Handwerker et al., 2004; Lindquist et al., 2009; Monti, 2011). The morphology of the HRF during human infancy has only been described in a single 3 month old infant following a large perinatal stroke, where a negative waveform was observed using a visual stimulus (Seghier et al., 2004). Of particular significance, key physiological parameters known to affect the HRF, including CBF and the cerebral metabolic rate of oxygen (CMRO_2_), show marked developmental changes during the perinatal period (Chen and Parrish, 2009; Chen and Pike, 2009a, 2010a; Miranda et al., 2006; Roche-Labarbe et al., 2011).

In this study we systematically characterized the ontogeny and morphology of the hemodynamic response to neural stimulation before and after the normal time of birth using an event-related experimental design and a somatosensory stimulus, and investigated the possible confounding effects of oral sedative medication by measuring global CBF. We hypothesized that the neonatal HRF differs from the canonical adult waveform; that there is a systematic maturational trend; and that application of an age-appropriate HRF in the analysis of fMRI data would significantly improve the identification of functional responses.

## Methods

The work was approved by the NHS research ethics committee, and written subject (or parental in the case of neonatal subjects) consent was obtained prior to all sessions of data acquisition.

#### fMRI study population

All neonatal subjects were recruited from the Neonatal Intensive Care Unit and Postnatal wards at the Queen Charlotte and Chelsea Hospital, London, UK during a period of 18 months between 2010 and 2011. A total of 19 preterm infants and 22 infants at term equivalent Post-Menstrual Age (PMA) were scanned. Data sets were excluded from the analysis if the sequence was not able to be completed (due to the subject waking), or due to excessive motion. The final study group (see Table 1) therefore consisted of 10 preterm infants scanned at median 34 + 4 weeks PMA (range 32 + 3 to 35 + 3 weeks) (9 male; median age at delivery 33 + 2 weeks PMA (26–34 + 3 weeks); median weight 1890 g (1560–2360); and median head circumference (HC) 30.07 cm (28–33)); and 15 infants scanned at term equivalent PMA (median age at scan 41 + 1 weeks PMA (38 + 1 to 44 + 0 weeks)) (5 male; median age at delivery 34 + 1 weeks (26 + 3–41 + 1); median weight 3035 g (2385–4770); median HC 35 cm (31–36.8). 12 of the infants studied at term equivalent age had previously been born prematurely. In addition, 10 healthy adult volunteers (median age 31.5 years (22–54 years), 5 male, all right-handed) were scanned using the same sequence and stimulation paradigm as those used in the neonatal subjects. Clinical details including antenatal, birth and postnatal care were recorded for each infant subject, and a detailed neurological assessment was carried out on all term-corrected age subjects by an experienced practitioner (Mercuri et al., 2005). Infants with extensive intraventricular hemorrhage on cranial ultrasound examination (grade 3 with ventricular dilatation, or grade 4 with parenchymal involvement), a history of poor condition at birth who had required vigorous neonatal resuscitation, other focal intracerebral lesions, hydrocephalus, congenital brain malformations or diagnosed metabolic disorders were excluded from the study group. Oral sedation (chloral hydrate 30–50 mg/kg dose) was administered approximately 20 min before scanning to 13 of the 15 term PMA infants, but to none of the premature infants. There were no adverse incidents during the data acquisition period.

#### fMRI image acquisition

MR imaging was performed on a Philips Achieva 3-Tesla system (Best, Netherlands) with an eight channel phased array head coil. All infants were assessed by a pediatrician prior to the scan, and the infants' temperatures, oxygen saturations and heart rates were monitored throughout the scan (Merchant et al., 2009). Ear protection was used in all infants (dental putty and adhesive ear muffs (Minimuffs, Natus Medical Inc, San Carlos, CA, USA)), and the head was immobilized using a polystyrene bead filled pillow from which the air was evacuated. High resolution T2-weighted images and 3D MPRAGE T1-weighted images were acquired for all infants and reviewed by a Neonatal Neuroradiologist (sequence parameters are detailed in Merchant et al., 2009).

fMRI data was acquired with a single shot echo-planar imaging (EPI) sequence lasting 8 min and 37 s (parameters: (TR) 500 ms; (TE) 45 ms; (flip angle) 90°; (matrix) 64 ∗ 64; (resolution(x ∗ y ∗ z)) 3.125 ∗ 3.125 ∗ 4 mm, total 1000 volumes). A relatively short TR was chosen (with the trade-off of decreased spatial resolution) as our goal was to characterize the BOLD signal HRF, and a faster sampling rate has been shown to be important when characterizing the HRF waveform particularly with respect to identifying the time to onset (Handwerker et al., 2004). To allow for this improvement in temporal resolution, whole-brain images could not be acquired, and therefore only 6 axial slices were acquired with the field of view placed above the level of the corpus callosum to give coverage of the peri-rolandic cortex. An identical scan protocol was used for both the adult and neonatal subjects.

#### fMRI experimental design

An event-related experimental design was used to acquire a sampled BOLD HRF following a brief (1 s) stimulus during which the subject's right hand was moved passively. It has previously been shown that robust changes in BOLD contrast can be identified and used to characterize the HRF using stimuli as brief as 0.1–0.3 ms (Hirano et al., 2011; Yesilyurt et al., 2010). To ensure full recovery of the BOLD signal to baseline, a 40.5 s inter-stimulus interval was used, during which time the BOLD signal was sampled every 500 ms. In the 1000 volumes acquired, a total of 12 complete stimulation and rest epochs were presented. A somatosensory stimulus synchronized to the image acquisition, was elicited with a programmable hand interface, consisting of a tailor-made inflatable balloon composed of 2 layers of latex around a nylon mesh, a control box and customizable software (Labview v8.1 2009, National Instruments, Austin, TX USA) (full description of stimulus device are detailed in Arichi et al., 2010). The balloon was sized and placed into the right hand of each subject; inflation of the balloon resulted in passive extension of the fingers, while deflation allowed flexion. Balloons of different sizes were used for the neonatal and adult subject groups, and the amplitude of balloon inflation was adjusted appropriately for hand size. It has been confirmed that the device is MR safe and fMRI compatible (Gassert et al., 2008) and has previously been used to demonstrate functional responses in groups of preterm and term infants (Arichi et al., 2010).

#### fMRI data analysis and HRF fitting

Data was analyzed using tools implemented in the FMRIB Software library (FSL, Oxford, UK, www.fmrib.ox.ac.uk/fsl) (Smith et al., 2004). Each functional data set was first visually examined for excessive motion artifact and image distortion, and data sets were discarded accordingly. If the motion was found to be isolated to a particular time period during the acquisition, the blocks of data affected by motion were then removed from the analysis, as systematic but false correlations in fMRI data are seen as a result of motion artifact despite standard registration and motion estimate regression techniques (Power et al., 2012). In particular, particular attention was placed on removing motion artifact which was associated specifically with the timing of the stimulus, which would have markedly affected the analysis and later model fitting. The remaining contiguous blocks of data were only included in the final analysis if greater than 40% of the entire data acquisition remained (representing a minimum of 5 peristimulus epochs).

Data was first processed using FEAT (fMRI Expert Analysis Tool, v5.98) and standard pre-statistics processing steps were applied: motion correction (using MCFLIRT (FSL's intra-modal motion correction tool), slice-timing correction, non-brain tissue removal, spatial smoothing (FWHM 5 mm), global intensity normalization and highpass temporal filtering (cut-off 50 s)) (Jenkinson et al., 2002; Woolrich et al., 2001). Head motion parameters were not included as confound regressors in the analysis, as additional data de-noising was performed using MELODIC (Model-free FMRI analysis using Probabilistic Independent Component Analysis (PICA, v3.0) (Beckmann and Smith, 2004)). Independent components assessed by their spatial representation and frequency power spectrum to represent physiological noise or motion artifact were filtered from the data prior to further statistical analysis. Time-series statistical analysis in FEAT was carried out using FMRIB's improved linear model (FILM) with local autocorrelation correction (Woolrich et al., 2001). A general linear model (GLM) was used to define the observed data using a convolution of the experimental design and an optimal basis set representing a dispersion range of possible HRF waveforms generated using FLOBS (FMRIB's linear optimal basis sets, v1.1) (Woolrich et al., 2004). For the generation of the basis set, this approach utilized a pre-specified range of parameters (in this case allowing for a greater range in the delay and height of the HRF than that typically seen in adults) to randomly generate possible HRF waveforms, from which principal component analysis was then used to identify an “optimal” basis set of 3 functions which maximally spanned a constrained HRF subspace of sensible waveforms (Woolrich et al., 2004). Parameter estimates for each of the explanatory variables and basis functions were then convolved in the GLM, converted to a t-statistic image by dividing by the relevant standard error, and then to a z-statistical score image at a threshold of 2.3 with a corrected cluster significance level of p < 0.05.

**Fig. 1:**
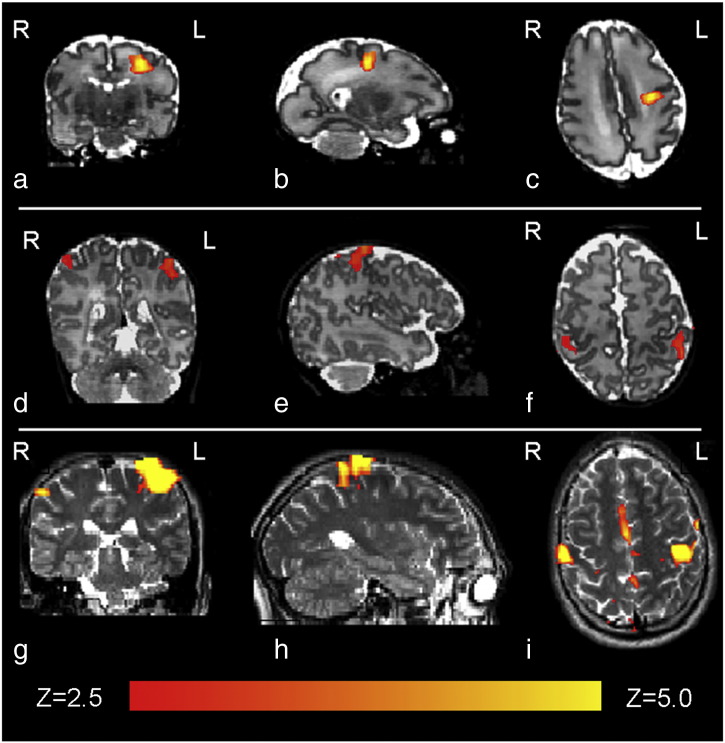
Identified clusters of functional activation following passive motor stimulation of the right hand, in a 32 + 3 PMA week preterm infant (top row: figures a,b,c); a term equivalent (PMA 41 + 1 weeks) infant (middle row: figures d,e,f), and a healthy 24 year old adult (bottom row: figures g,h,i). A thresholded statistical map with a corrected cluster significance of p < 0.05 has been overlaid on the subject T2-weighted image.

The BOLD signal time-series was extracted and averaged from a region of interest (ROI); defined as voxels above the 90th centile in z-score within the cluster of activation in the contralateral primary somatosensory cortex identified with the GLM analysis and the complete fit of the data derived from an F-test of the parameter estimates from the individual basis functions (Fig. 1). The time-series was averaged across the peristimulus period and then converted to a percentage signal change (relative to the baseline, defined as the time-points across the 2 s prior to stimulus onset). For each individual subject and the group analysis, the converted peristimulus data was then fitted with a double gamma distribution function (robust non-linear least squares fit, trust-region algorithm) to model a subject-specific and population age specific HRF using the curve-fitting toolbox implemented in MATLAB (2009b, The Mathworks, Natick, MA USA). The use of two gamma distribution functions for modeling the HRF has been widely described in the literature, and has been found to provide a reasonable characterization of all of the key positive (positive peak) and negative (initial dip and undershoot) features of the HRF (Boyton et al., 1996; Friston et al., 1998).

### Global CBF estimation

Animal and adult fMRI work have demonstrated that changes in the HRF peak amplitude and time to peak can be artificially induced by the experimental manipulation of baseline CBF (Chen and Parrish, 2009; Chen and Pike, 2009a; Cohen et al., 2002; Colonnese et al., 2008). To investigate if the administration of chloral hydrate could be responsible for any observed differences in HRF morphology, global CBF was measured from a separate cohort of 14 healthy term born infants who were then subdivided into two groups (those who were sedated with low dose chloral hydrate medication (30–50 mg/kg/dose) prior to scanning, and those who were not) (see Table 2). Infants who had required neonatal resuscitation or had any abnormalities (as described above in the fMRI study population) were ineligible for this study. The infants were paired by PMA at the time of scan, as it has previously been shown that CBF increases in the months following delivery (Greisen, 1986, Varela et al., 2012).

Cerebral blood flow measurement data was acquired using an optimized Phase Contrast Angiography (PCA) sequence (Varela et al., 2012). A multi-slice inflow arteriogram ((TR) 21 ms; (TE) 6 ms; (matrix) 160 ∗ 132; (resolution(x ∗ y ∗ z)) 1 ∗ 1 ∗ 1 mm) was performed for geometrical planning of the PC flow measurement sequence. The acquisition plane was positioned at the level of the sphenoid bone, where the internal carotid and basilar arteries are approximately parallel and simultaneous flow measurements can be done using a single imaging plane and encoding velocity along the through-plane direction (Buijs et al., 1998; Varela et al., 2012). Flow data was acquired using a sequence optimized for neonatal subjects ((TR) 7 ms; (TE) 4.2 ms; (flip angle) 10^o^; (resolution(x ∗ y ∗ z)) 0.6 ∗ 0.6 ∗ 4.0 mm; (maximal encoding velocity (v_ENC_)) 120 cm/s) (Varela et al., 2012). Instantaneous flux was measured for each cardiac phase and artery, using a time-resolved ROI method (Q-flow Philips image analysis package, release 2.3.5.0 (Philips Corporation, Best, Netherlands)). The mean velocity across the ROI was multiplied by vessel area to give an estimate of instantaneous flux, and flow in the vessel calculated from the mean of the instantaneous flux across the cardiac cycle. Total flow to the brain was obtained by summing blood flow in the two internal carotid arteries and the basilar artery. Whole brain volume was measured from high resolution T2-weighted images, following tissue segmentation using in-house software. Each subjects’ T2-weighted images were first bias-field corrected using FAST v4.1 (FMRIB's automatic segmentation tool (Zhang et al., 2001)). The corrected image was then aligned to a 4D neonatal atlas using non-linear registration as implemented in IRTK (Image Registration Toolkit; www.doc.ic.ac.uk/~dr/software/) (Kuklisova-Murgasova et al., 2011; Rueckert et al., 1999). The CSF and extra-cerebral tissue was subtracted from the segmented image, and then whole brain volume was then computed in mm^3^. Global CBF in ml/100 g/min was then calculated by dividing the total flow to the brain by the brain volume with a further correction for brain density (1.05 g/ml in neonates) (Delpy et al., 1987).

### Application of population specific HRF models to experimental data

To test the value of the HRF waveforms derived in the main study, they were applied into the GLM analysis of 6 preterm (median age 34 + 0 weeks PMA (32 + 2–34 + 5) and 6 term equivalent (median age 40 + 5 weeks PMA (39 + 0–43 + 3) infant data sets from a previously collected study group (Arichi et al., 2010). A block experimental design had been utilized with periods of somatosensory stimulation lasting 24 s, interleaved with rest periods of 24 s (image acquisition and experimental design described in Arichi et al., 2010). The experimental design was convolved with the age-specific HRF waveform into the GLM for analysis, using the parameters derived from the HRF characterization studies. Standard pre-processing steps and data analysis (as described previously) were performed using FEAT v5.98, and z-statistical score images were generated with a threshold of 2.3 and corrected cluster significance of p < 0.05. Each of the individual subject statistical maps were then registered to a custom-made neonatal template for higher level analysis using linear registration (Arichi et al., 2010; Jenkinson et al., 2002; Smith et al., 2004). A fixed-effects model was then applied to identify group means, and perform a paired t-test on the lower-level statistical images.

## Results

### HRF characterization

Following passive motor stimulation of the right hand lasting 1 second, clusters of functional activation were identified in the contralateral (left) primary somatosensory cortex in all 3 subject groups (Fig. 1). As observed in previous work and in the developing rat brain, a trend towards co-activation of the ipsilateral primary somatosensory cortex and associated sensori-motor areas such as the supplementary motor area was seen with increasing age (Arichi et al., 2010; Colonnese et al., 2008). To maintain consistency across subject groups, HRF characterization was therefore performed using the BOLD signal time-series from a region of interest (ROI) in the contralateral cortex only.

**Fig. 2:**
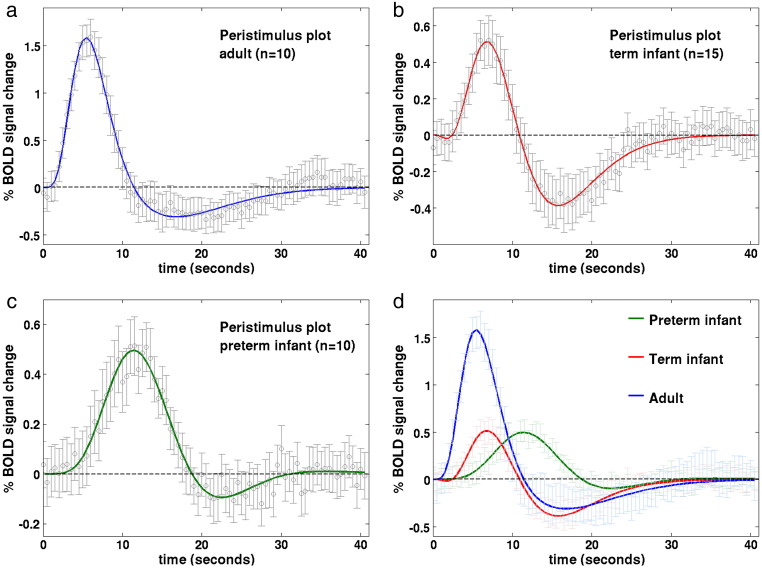
Peristimulus timeseries plots for the (a) adult; (b) term equivalent infant; (c) preterm infant groups. Stimulation occurred at time point 0, lasting a total of 1 s. The mean % BOLD signal change (relative to the pre-stimulus signal) at each timepoint (circles) is shown fitted with a double gamma probability distribution function. Error bars represent 2 SEM. (d) A decrease in the time to peak of the HRF, and an increase in peak amplitude is seen with increasing age.

**Fig. 3:**
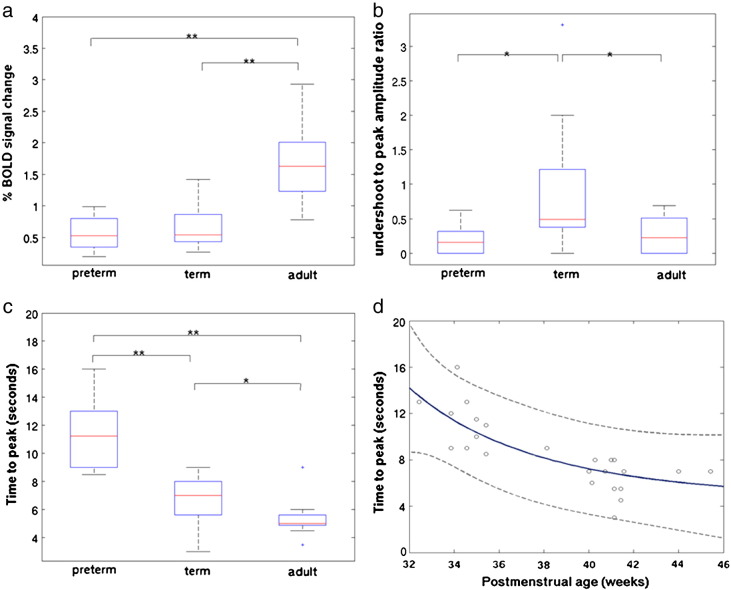
(a) A significant difference was seen between both the preterm and term infant groups and adult subjects in the amplitude of the HRF positive peak. (Boxplots: box represents 25th and 75th centiles and central line the group median; outliers denoted by ‘+’ symbol; Mann–Whitney–Wilcoxon test with Holm–Bonferroni correction for multiple comparisons, p < 0.05*, p < 0.01**)). (b) The ratio of the negative HRF undershoot to the amplitude of the positive peak was significantly deeper in the term infant group in comparison to both the preterm and adult groups. (c) A significant maturational trend towards a reduction in the time taken to achieve the positive HRF peak was identified across the three patient groups. (d) In the neonatal subjects only, an inverse exponential relationship between increasing post-menstrual age (in weeks) and the time to the HRF peak (in seconds) was identified (r^2^ = 0.6479; dashed lines represent 95% population confidence intervals).

A clear developmental trend in the shape parameters of the HRF was identified, characterized by a reduction in the time to positive peak and an increase in positive peak amplitude with increasing age (Table 3). In the adult group, the parameters and morphology of the sampled HRF waveforms were in agreement to those described in the literature (Glover, 1999; Handwerker et al., 2004); with a median time to the positive peak of 5.38 seconds (range 4.5 to 9), a median peak amplitude of 1.63 % signal change (range 0.78 to 2.93) (relative to the pre-stimulus baseline BOLD signal), and median positive peak to undershoot ratio 0.23 (range 0 to 0.69) (Fig. 2a). In comparison, the HRF waveform in the term equivalent post-menstrual age (PMA) infant group (Fig. 2b) was found to have a significantly longer time to peak of 7.0 seconds (range 3 to 9) (p < 0.05: Mann–Whitney–Wilcoxon test, Holm–Bonferroni correction for multiple comparisons), with a significantly smaller peak amplitude peak of 0.54% signal change (range 0.27 to 1.42) (p < 0.01) (Fig. 3a), and significantly deeper negative undershoot period with a ratio to the positive peak of 0.49 (range 0 to 3.31) (Fig. 3b). The median positive peak amplitude (0.52%, range 0.19 to 0.99) of the preterm infants was similar to those of the term infants (p = 0.5235), although a proportionately shallower undershoot period was seen (ratio 0.15 (range 0 to 0.62)) (Fig. 2c). A significant lengthening in the median time to peak at 11.25 s (range 8.5 to 16) was seen in the preterm infants in comparison to both the adult and term infant groups (p < 0.01) (Fig. 3c). An inverse exponential trend was observed with increasing PMA associated with a decrease in the time taken to reach the positive peak of the HRF (Fig. 3d). The median ratio of the undershoot to positive peak amplitude was significantly different between the term infant group with both the preterm (p < 0.05) and adult groups (p < 0.05) (Fig. 3b). There was no significant difference between the adult group and preterm infant group in the undershoot to positive peak ratio (p = 0.8331).

### Global cerebral blood flow estimation

Global CBF data was acquired from a total of 14 term born infants who were then subdivided into two paired groups (those who were sedated for scanning and those who were not) by postmenstrual age at the time of scan. There were no significant differences between the two groups in the age of the infants at scan (Mann–Whitney–Wilcoxon test: p = 0.5198); the weight (Wilcoxon signed rank test: p = 0.3750); occipito-frontal head circumference (p = 0.8438); or brain volume (p = 0.1562). No significant difference was identified in the global CBF between the paired sedated (median: 22.40 ml/100 g/min) and unsedated groups (median: 20.78 ml/100 g/min) (Wilcoxon paired signed rank test: p = 0.4688). These CBF values are in good agreement with those previously described using diverse measurement techniques (Edwards et al., 1988; Greisen, 1986; Varela et al., 2012).

### Application of population specific HRF models to experimental data

**Fig. 4:**
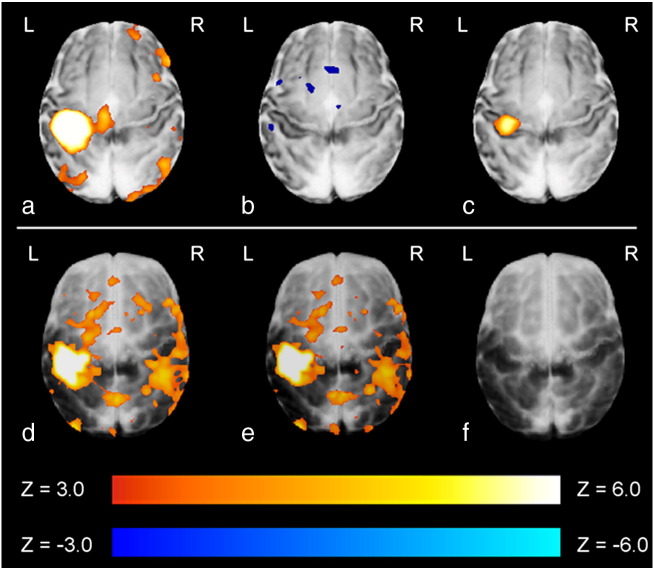
*Preterm infant group (top row):* (a) A large cluster of positive activation was identified in the contralateral somatosensory cortex when an age-specific HRF model was convolved into the GLM analysis in a group of 6 preterm infants; (b) this was not seen when the analysis was repeated using the canonical adult t-test analysis was performed on the statistical maps derived from the lower level analyses. *Term equivalent infant group (bottom row):* (d) Significant clusters of functional activity were identified when both age-specific and (e) canonical adult HRF models were convolved into the GLM analysis of 6 infants at term corrected PMA; (f) There was no significant difference between the two forms of analysis on a paired t-test analysis.

**Fig. 5:**
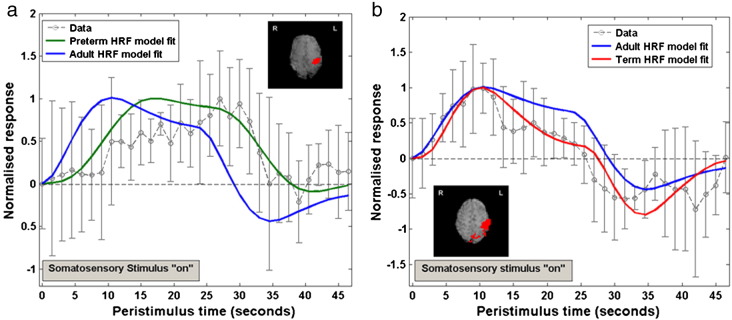
Example peristimulus timeseries data derived from clusters of activation (inset pictures, red) identified following passive motor stimulation of the right hand. (a) In a preterm infant, the age-specific HRF can be seen to greatly improve the model fit (green), as the peak in contrast occurs much later than would be predicted using the adult HRF (blue). (Error bars represent 1SD from the mean) (b) In an infant at term corrected PMA, the age-specific HRF improves the model fit (red), by incorporating the deeper undershoot period seen following the positive peak.

To test the value of the derived HRF models the empirical waveforms were then convolved into the GLM analyses of data collected from 12 further infants, using a block paradigm of somatosensory stimulation (Arichi et al., 2010). In 6 preterm subjects, a fixed effects GLM analysis following convolution of a preterm age-appropriate HRF waveform into the lower level subject analyses identified a large but well localized cluster of positive signal functional activation in the primary somatosensory cortex contralateral to the side of stimulation (left hemisphere) (Fig. 4a). In contrast, when the same analysis was performed with convolution of the empirical adult HRF, only small areas of negative signal change were identified in the left peri-rolandic region, with no significant areas of positive signal activation (Fig. 4b). In agreement with these findings, a direct comparison of the two types of analysis (paired t-test on the effect-size estimates) identified a significant and well localized cluster in the left primary somatosensory cortex (Fig. 4c). This difference can be seen in an exemplar study (Fig. 5a) where convolution of the experimental design with the preterm infant HRF is shown to markedly improve the model fitting to the BOLD signal data from the identified cluster of activation with correlation coefficient 0.8407 and sum of squared errors (SSE) 1.9013, in comparison to the canonical adult HRF (correlation coefficient 0.3496, SSE 8.0755).

This process was repeated in a group of 6 infants at term corrected PMA on whom data had been collected using an identical experimental paradigm. In an exemplar study (Fig. 5b) convolution with the term infant HRF waveform can be seen to improve the fit to the data with a correlation coefficient of 0.9096 and SSE 1.5775, in comparison to 0.9055 and SSE 3.0254 using the canonical adult HRF. Convolution of the term infant derived HRF and adult subject derived HRF with the experimental model identified similar clusters of positive functional activation most significantly in the left somatosensory cortex, but with co-activation of the ipsilateral right somatosensory cortex (Figs. 4d,e). A paired t-test did not identify any significant areas of difference between the two forms of analysis (Fig. 4f).

## Discussion

Using a combination of optimized fMRI scanning parameters, an appropriate and precise somatosensory stimulus, and an event-related experimental design, we have been able to characterize the morphology of the BOLD contrast HRF waveform in the developing human brain. As described in the rat brain, a systematic maturational change in the morphology and parameters of the HRF was seen (Colonnese et al., 2008), both in terms of the time-to-peak and overall magnitude of the response. In addition, we provide data showing that at term corrected PMA, global CBF is unchanged by low-dose pharmacological sedation suggesting that the observed differences cannot be ascribed to the use of sedation but are secondary to developmental changes in cerebro-vascular physiology. The potential improvements in accuracy yielded from the use of an age-appropriate HRF model convolved into the GLM analysis are demonstrated in two infant groups, with a significant effect seen when applied to preterm infant functional data.

### Developmental changes in neurovascular coupling

In comparison to the canonical form seen in the mature adult brain, the amplitude of the HRF positive peak was found to be significantly less in the developing neonatal brain. In addition, the time taken to attain the positive peak amplitude of the HRF was found to decrease significantly with increasing age. The physiological reasons underlying these differences are likely multi-factorial, and involve many stages of the neurovascular coupling cascade which ultimately culminates in the hemodynamic changes responsible for the BOLD contrast response (Cauli and Hamel, 2010; Harris et al., 2011). Due to limitations inherent to studying the in-vivo human infant brain, the effects of developmental changes on these processes have not been extensively investigated; and many of the detailed measures common to calibrated fMRI experiments in adult subjects and animal models are not applicable to this population (Gaillard et al., 2001).

Of note, robust electrophysiological responses to simple somatosensory stimuli can be elicited at a significantly younger age than reliable BOLD signal responses have been described in both animal and human subjects (Vanhatalo and Lauronen, 2006). Although it is unlikely that neural activity in very immature subjects is occurring without the vascular provision of the required metabolic substrates, it does suggest that marked differences in the dynamic coupling of the neural activity and vascular response must underlie some of the trends identified in this study. The neurovascular coupling cascade is thought to involve multiple signaling pathways encompassing perivascular astrocytes, vasoactive chemical agents, and direct neuronal connections (Cauli and Hamel, 2010; McCaslin et al., 2011). Changes in astrocyte-mediated processes may be of particular significance as animal studies have found marked increases in number, size and local connectivity at an age which corresponds to the human age groups studied in this work (Harris et al., 2011; Kaur et al., 1989).

#### Trends in cerebral hemodynamics in early human development

A localized increase in CBF is known to be the key to the positive peak of the BOLD response through the change in signal which results from an increase in local oxygenated hemoglobin (Hb) (Buxton et al., 2004; Chen and Pike, 2009a, 2009b; Hillman et al., 2007). Arterial Spin Labeling (ASL) experiments have demonstrated that the local CBF time course following stimulation closely mirrors that of the BOLD HRF, and furthermore have suggested that a feedback mechanism may contribute to a post-stimulus suppression in CBF which correlates with the HRF undershoot (Chen and Pike, 2009a, 2009b). Global decreases in CBF following caffeine administration have been shown to lower the baseline BOLD signal, increase the percentage signal change of BOLD responses and shorten the time to peak (Chen and Parrish, 2009; Liu et al., 2004; Perthen et al., 2008); while increases in CBF caused by the cerebral vasodilating effects of carbon dioxide have been shown to result in the converse (Chen and Pike, 2010a, 2010b; Cohen et al., 2002). These alterations are known to occur in the context of unchanged neurophysiological and metabolic parameters (Chen and Pike, 2010a; Matsuura et al., 2000) and are therefore thought to be primarily due to the linking of arteriolar compliance as a function of baseline CBF (Liu et al., 2004).

Global CBF is known to increase dramatically during early human development, with preterm infant brain values approximately half that of a full term infant, with a further twofold increase in adult life (Edwards et al., 1988; Greisen, 1986; Roche-Labarbe et al., 2011; Varela et al., 2012). Given that such a systematic rise in baseline CBF would be expected to induce HRF changes similar to hypercapnia, the observed trends are therefore likely secondary to developmental changes in the capacity of the local arterioles to increase local CBF through the neurovascular coupling cascade. This would be in keeping with histological studies which suggest that the human fetal cortical microvasculature develops radially from the superficial leptomeningeal vessels, with muscularization of the extrastriatal arterioles and capillary beds not established until close to term equivalent PMA (Kamei et al., 1992; Kuban and Gilles, 1985; Norman and O'Klusky, 1986). Moreover, cerebral vessel density and volume has been shown to approximately double from the newborn to adult primate cortex, with the bulk of this change occurring at the capillary level, which may also translate to a faster and higher amplitude local CBF response (Risser et al., 2009).

Near-Infrared Spectroscopy (NIRS) studies have shown that quantitative measures of cerebral blood volume (CBV) remain unchanged both throughout the preterm period and during the first weeks after full term gestation (Franceschini et al., 2007; Roche-Labarbe et al., 2011; Wyatt et al., 1990). This would suggest that the empirical steady-state relationship (Grubb's power law) between whole brain CBV and global CBF seen in adults may differ or does not have a constant exponent during the preterm and neonatal period (Buxton et al., 2004; Grubb et al., 1974). A constant CBV coupled to an increasing CBF through the late preterm to term infant period would lead to a shortening in the mean transit time (MTT) of oxygenated Hb; as the Stewart-Hamilton principle states that CBV can also be represented as the product of CBF and MTT (Elwell et al., 1997; Meier and Zierler, 1954). A change in MTT may therefore in part explain the shortening of the time taken to achieve the positive peak of the HRF seen with increasing PMA.

A proportionately deeper post-stimulus undershoot was seen in the term infant subjects, despite a similar positive peak amplitude throughout the neonatal period. It has been suggested that the undershoot period may reflect a transient increase in deoxygenated-Hb due to a temporal mismatch between the CBF and draining venous CBV response due to differences in vessel wall compliance (Buxton et al., 1998, 2004; Chen and Pike, 2009b). In the context of established biomechanical models such as the Balloon Model, a deep post-stimulus undershoot can be explained by an initially stiff post-capillary-bed venous compartment which becomes compliant after prolonged expansion, leading to the volume outflow of the system resembling a hysteresis loop (Buxton et al., 1998, 2004). Alternatively, there is also recent evidence to suggest that transient decoupling between the CBF and a sustained post-stimulus increase in the local cerebral metabolic rate of oxygen (CMRO_2_) results in deoxygenated Hb accumulation and therefore a decrease in BOLD signal (Dechent et al., 2011; Hua et al., 2011). In the neonatal brain, the latter effect may predominate as marked increases in neuronal density and integration occur in the late preterm to term infant period, and these changes are associated with a significant maturational rise in CMRO_2_ (Altman et al., 1988; Chugani and Phelps, 1986; Roche-Labarbe et al., 2011).

### The possible effects of sedative medication

In this study, induced sedation with chloral hydrate was given in the majority of the term infant subjects, who are more prone to motion and may become distressed during image acquisition in comparison to preterm infants. Although functional responses and patterns of resting state connectivity can be identified in naturally sleeping infants, the increased head motion inherent to these subjects may lead to the systematic identification of false patterns of functional activity (Power et al., 2012; Satterthwaite et al., 2012; Van Dijk et al., 2012). In contrast, sedation with chloral hydrate does not affect either the identification or topology of resting state networks in neonatal subjects (Doria et al., 2010). Further HRF characterization studies may be possible in unsedated infants if novel motion-resistant image acquisition and analysis techniques can be optimized.

Electrophysiological studies have shown that the amplitude and character of neural responses are not affected by mild to moderate doses of induced sedation (such as used in this study with chloral hydrate) (Avlonitou et al., 2011; Sisson and Siegel, 1989). It has also been suggested that sedative medication may alter baseline CBF, thereby explaining the inconsistent findings in previous infant fMRI studies (Lindauer et al., 1993; Rivkin et al., 2004; Seghier et al., 2006). We found that sedation did not affect global mean CBF in paired samples of healthy term infants. In the rat brain, correlated alterations in both the BOLD fMRI and local field potentials have been described to a somatosensory stimulus during urethane and alpha-chloralose anesthesia, suggesting that tight neurovascular coupling is preserved even during induced anesthesia (Huttunen et al., 2008). Although we cannot completely exclude a possible effect of chloral hydrate on local hemodynamics, animal data suggests that very high doses of the active compound 2,2,2-trichloroethanol act as an agonist of non-classical K^+^ channels in smooth muscle cells, increasing local CBF and leading to uncoupling with an unchanged CMRO_2_ (Parelkar et al., 2010; Uematsu et al., 2009). This effect would not be in keeping with the deep negative undershoot period observed in the sedated term infants.

#### Study design and further work

Although an event-related fMRI design with a widely-spaced constant inter-stimulus interval is relatively inefficient at both detecting activity and HRF estimation (Dale and Buckner, 1997; Handwerker et al., 2004; Murphy et al., 2007), it was chosen in this study as particular assumptions could not be made, in particular whether overlapping impulses would sum in a linear fashion (Bandettini and Cox, 2000; Buxton et al., 2004; Gu et al., 2005). Data was initially analyzed using a basis set to allow flexible HRF modeling; this approach is particularly suitable in subjects where alterations may occur due to physiological and/or clinical factors, although at the risk of fitting physiologically implausible HRF shapes leading to fewer degrees of freedom and a decrease in power (Monti, 2011; Steffener et al., 2010; Woolrich et al., 2004).

The term equivalent age study group consisted mostly of infants who had been born prematurely (12/15), and although previous work has suggested that the functional activity is well localized regardless of the gestational age at birth (Arichi et al., 2010), further work will be required to identify any more subtle effects on HRF morphology which may result from preterm birth. The somatosensory cortex was used as the substrate for this study, as robust responses are seen with a variety of imaging modalities including fMRI in both preterm and term neonates (Arichi et al., 2010; Erberich et al., 2006; Kusaka et al., 2011; Vanhatalo and Lauronen, 2006). HRF characterization studies in the adult brain have demonstrated that subtle differences exist in distinct brain regions using different stimulus types, and that significant differences are identified when this variation is incorporated into the GLM analysis (Handwerker et al., 2004; Miezin et al., 2000). Further work to characterize this inter-region variability will be of particular importance in the neonatal brain, as resting state fMRI studies have shown that different neural networks appear to develop at different rates; with the auditory system maturing before others (Doria et al., 2010).

#### Implications for future fMRI studies of neonatal subjects

The benefits of an age-appropriate HRF for convolution into the GLM have been demonstrated here in two example preterm and term infant groups. This effect was most marked in the preterm infant group, where a cluster of positive activation in the primary somatosensory cortex was only identified when an age-appropriate HRF was used in the GLM design model, incorporating the significantly longer time taken to achieve the positive peak. In the term infant group, a large area of positive functional activation was identified irrespective of the HRF model used, and a significant difference was not seen when comparing the age-specific and adult canonical HRF models. This is explained by the relative similarity in the time to the positive peak for the term infant and adult groups, which will lead to a similar positive overshoot time regardless of the proportionately deeper undershoot period. However, at shorter inter-stimulus intervals than used in this work, the rise rate and amplitude of BOLD signal change would be significantly reduced should the next period of stimulation occur during the undershoot (McClure et al., 2005). It is notable that no significant areas of negative BOLD response were identified in either subject group using an age-appropriate HRF model. Negative BOLD responses have been more commonly reported in later infancy (approximately 3 months of age and above) where it has been postulated that increasing neuronal energy demands exceed the available supply of oxygenated hemoglobin (Seghier et al., 2004; Yamada et al., 2000). The results of our work suggest that further systematic characterization of BOLD responses throughout childhood would be required before this hypothesis could be conclusively accepted.

## Conclusions

In summary, we provide characterization of the HRF in the healthy human brain before and around the normal time of birth, and demonstrate a developmental trend in early human HRF morphology similar to that seen in the rodent brain. Moreover, the data provide evidence that the marked changes in brain structure known to occur in the third trimester of human development are also accompanied by a sequence of maturation in the brain's hemodynamic responses to stimulation. These maturational changes are likely to be due to both probable developmental alterations in the underlying neurovascular coupling and known changes in cerebrovascular physiology. These findings demonstrate that BOLD fMRI responses can be reliably identified in neonatal subjects, and offer the potential to improve the accuracy of analysis in studies involving this population. Although the effects of sedation cannot be completely excluded, the described HRF parameters still remain relevant, and can be applied to much needed future fMRI studies in this vulnerable population.

## Author contributions

TA, NT, NM, SJC collected the data; TA, MV, SJC optimized the imaging sequences; AM, AA, EB designed and manufactured the stimulus; TA, GF, CFB analyzed the data; TA and ADE planned the study and wrote the manuscript.

## Conflicts of interest

The authors have no conflicts of interest to disclose.

## Figures and Tables

**Table 1:** Subjects included in the hemodynamic response function (HRF) characterization analysis.

Group	Number of subjects (male)	Post-menstrual age at scan (median, range)	Gestational age at birth (median, range)	Weight (median, range)	Head circumference (median, range)
Preterm infant	10 (9)	34 + 4 weeks (32 + 3–35 + 3)	33 + 2 weeks (26 + 0–34 + 3)	1890 g (1560–2360)	30.07 cm (28–33)
Term infant	15 (5)	41 + 1 weeks (38 + 1–44 + 0)	34 + 1 weeks (26 + 3–41 + 1)	3035 g (2385–4770)	35 cm (31–36.8)
Adult	10 (5)	31.5 years (22–54)	n/a	Not recorded	Not recorded

**Table 2:** Infant subjects included in the global cerebral blood flow (CBF) estimation analysis.

Group	Post-menstrual age at scan (median, range)	Weight (median, range)	Head circumference (median, range)	Brain volume (median, range)	Global cerebral blood flow (median, range)
Sedated (n = 7)	41 + 3 weeks (38 + 1–43 + 4)	3700 g (3115–3920)	35.5 cm (34–36.5)	400.8 ml (368.0–444.0)	22.40 ml/100 g/min (19.15–26.78)
Unsedated (n = 7)	40 + 3 weeks (38 + 4–43 + 0)	3500 g (2652–3944)	35.5 cm (34–36.7 cm)	388.0 ml (324.0–446.5)	20.78 ml/100 g/min (19.20–28.87)

**Table 3:** Measured parameters of the hemodynamic response function in the 3 subject groups. (*p < 0.05, **p < 0.001 Mann–Whitney–Wilcoxon test in comparison to adult group, Holm–Bonferroni correction for multiple comparisons).

Group	n	Time to positive peak (median, range; seconds)	Positive peak amplitude (median, range; % BOLD signal change)	Undershoot to positive peak ratio (median, range)
Preterm infant	10	11.25 (8.5–16)**	0.52 (0.19–0.99)**	0.15 (0–0.62)
Term equivalent infant	15	7.0 (3–9)*	0.54 (0.27–1.42)**	0.49 (0–3.31)*
Adult	10	5.38 (4.5–9)	1.63 (0.78–2.93)	0.23 (0–0.69)
